# Juvenile European hedgehogs (*Erinaceus europaeus*) at rescue centers and their release rate depending on their weight on admission

**DOI:** 10.1371/journal.pone.0258273

**Published:** 2021-10-07

**Authors:** Gabriela Lukesova, Eva Voslarova, Vladimir Vecerek

**Affiliations:** Faculty of Veterinary Hygiene and Ecology, Department of Animal Protection and Welfare and Veterinary Public Health, University of Veterinary Sciences Brno, Brno, Czech Republic; University of Veterinary Medicine Vienna: Veterinarmedizinische Universitat Wien, AUSTRIA

## Abstract

This study aimed to assess the numbers of juvenile European hedgehogs admitted to rescue centers in the Czech Republic from the viewpoint of their weight on admission, the reason for their admission, and the success rate of their release back into the wild. The results of our study show varying levels of success in the rearing of hedgehogs admitted at different ages (weights) and a varying period required for their rehabilitation. The greatest chance of release was seen in hedgehogs with a weight on the admission of 500–599 g (64.22% released) and 400–499 g (63.31% released). In contrast, the smallest number of young hedgehogs successfully rehabilitated and released was seen in hoglets weighing 200–299 g (35.24% released) on admission, which corresponds to the weight of hedgehogs at the time of weaning. Time spent at a rescue center may pose an undesirable threat to the lives of animals in some categories. Hedgehogs weighing up to 99 g on admission spent the longest period time at rescue centers (a median of 48 days), while hedgehogs weighing 500–599 g on admission spent the shortest time (a median of 7 days). The majority of hedgehogs in the lowest weight categories were admitted due to their inability to survive on their own. A large percentage of hedgehogs of greater weight, in contrast, were juvenile hedgehogs brought to rescue centers needlessly. The percentage of released animals did not exceed 65%, however, even for entirely independent categories of older juveniles. From this perspective, the fact that hedgehogs are often brought to rescue centers in the belief that they are not self-sufficient young, though they are actually juvenile or even adult individuals that do not require human care, can be considered a significant finding.

## Introduction

The European hedgehog (*Erinaceus europaeus*) is one of two hedgehog species occurring in the Czech Republic [1]. Hedgehogs often live in the vicinity of towns and villages as these provide greater protection against predators including badgers and foxes that tend, in contrast, to avoid these places [2]. Hedgehogs also like to use gardens and parks in towns and villages to search for their food [3], which is comprised mainly of invertebrates. The public is often aware of the fact that hedgehogs occur in their vicinity and that they can support the presence of hedgehogs in their gardens by feeding them, by making gardens accessible, or by playing a part in their protection in other ways. Anthropogenic activity may also harm hedgehogs in the form of mortality on the roads [4], predation by domestic animals [5], habitat fragmentation [6] and a possible habitat loss resulting from greater use of land for agricultural purposes [7]. These factors contribute widely to the mortality of individuals, have a negative effect on populations and pose a threat to many animal species [8].

Hedgehogs are one of the most frequently admitted mammals in European rescue centers as documented by e.g. Kirkwood [9] in Great Britain and Molina Lopéz [10] in Spain. Also in the Czech Republic, European hedgehogs (most often young) are frequently admitted to rescue centers but only about half of the young can be subsequently released back into the wild [11]. Similarly, young make up a large proportion of admitted hedgehogs in Great Britain [12] or Portugal [13]. Since European hedgehogs inhabit a large part of Europe, their breeding season may vary in different areas depending on the climatic conditions. The breeding season is also closely associated with the length of hibernation, which also depends on the climate. Hibernation is shorter in warmer areas and the breeding season begins earlier and ends later [14] meaning that females may have two litters in a single year [15]. In colder areas, in contrast, hibernation is longer and ends later, and just a single litter a year is usual [16]. In some parts of Europe, such as Britain, there is also speculation about the possibility of two litters. Another possibility is that young born later than usual is young from a later replacement litter of a mother that has mated again after losing the young from a previous litter [17]. The mating period differing under different climatic conditions is also confirmed by the fact that female hedgehogs are only receptive to mating when the climatic conditions are suitable [18] as this is associated with a greater chance of survival of the young. The mortality rate is generally high in young hedgehogs. Morris [19] reported mortality of as much as 20% in a litter, resulting primarily from the choice of an unsuitable birth den or the unsuitable timing of birth. Rasmussen et al. [20] reported 70% of hedgehogs surviving their first year of life in residential areas. Hibernation is another critical period in the life of a hedgehog, and preparing properly for the first hibernation is a critical task for young animals. Later-born young may seem to have a lower chance of surviving hibernation due to insufficient weight, though this theory has been refuted by Bunnell [17]. It is, according to this author, possible that young born at a later time of year do not have a lower chance of successful hibernation because they put on weight faster than hedgehog young born earlier. According to Yarnell et al. [21], even the release of young in the winter does not reduce their chances of survival. The ideal weight given in the studies differs. Jensen [22], for example, considers a minimum of 513 g the ideal weight before the first hibernation. The determination of age in hedgehogs is complicated by not entirely clear indicators, though certain physical parameters, such as the length of the body, the limbs and the jaw, can be considered suitable parameters [23].

This study aimed to analyse the number of juvenile European hedgehogs admitted to rescue centers in the Czech Republic in the period 2010 to 2019 and to assess the success of their rearing and release back into the wild depending on their weight measured on the admission of these juvenile hedgehogs to rescue centers.

## Materials and methods

We obtained data on juvenile European hedgehogs admitted to rescue centers from the database of the Ministry of the Environment, which coordinates the work of rescue centers in the Czech Republic. This database included information on juvenile European hedgehogs admitted to 34 rescue centers falling under the National Network of Rescue Centers of the Czech Republic in the period from 2010 to 2019. The subject of the evaluation was data relating to the numbers of juvenile European hedgehogs admitted, their weight, and the dates on which they were admitted to and left the rescue centers. Since the age of the hoglets admitted to rescue centers was usually unknown, it was only estimated based on some physical indicators by a veterinarian during the admission process. Only data on individuals recorded as hoglets/juvenile hedgehogs were analysed in our study.

For evaluation, the animals were divided into groups by the year in which they were admitted and the month in which they were admitted to rescue centers. The juvenile hedgehogs whose weight at the time of admission to rescue centers was given were divided into groups of animals weighing up to 99 g, 100–199 g, 200–299 g, 300–399 g, 400–499 g, 500–599 g, 600–699 g and 700–799 g. The number of hedgehogs in each weight category that was released back into the wild and the length of time they spent at the rescue center was calculated in the individual weight categories. Evaluation of the length of time spent at rescue centers independence on weight was performed only for those individuals for which weight on admission, date of admission to the rescue center and date on which their stay at the rescue center came to an end were stated. The median length of stay for the individual weight categories was calculated from this data. The maximum, minimum, median and average period spent in days was also determined in individual weight categories for hedgehogs that were released back into the wild following their stay at the rescue center. The percentage of hedgehogs taken to rescue centers because they needed human care due to being orphaned, due to starvation, or having been late-born young, due to injuries (caused by dogs, cats, other animals, cars or garden equipment), infectious or parasites, fall into pits, and hedgehogs captured needlessly (as determined by a veterinarian) or with other reasons (entanglement, intrusion or unknown reasons) were also studied in the individual weight categories.

The data were evaluated in the statistical program UNISTAT 6.5 for Excel (Unistat Ltd., London, UK). Spearman’s coefficient, according to which a rank correlation coefficient was determined, was used to assess development in the number of juvenile European hedgehogs admitted by year and to determine the relationship between the period spent at rescue centers and the weight category. A Fisher exact test using the 2x2 contingency table methodology was used for the statistical evaluation of differences in frequencies among groups of hedgehogs admitted in different months and the frequencies among groups of hedgehogs according to their weight. Kruskal-Wallis ANOVA (and multiple comparisons for t-distribution) was used for evaluation of the period spent at rescue centers for individual weight categories. A value of p < 0.05 was determined as statistically significant in all tests used.

## Results

**Fig 1:**
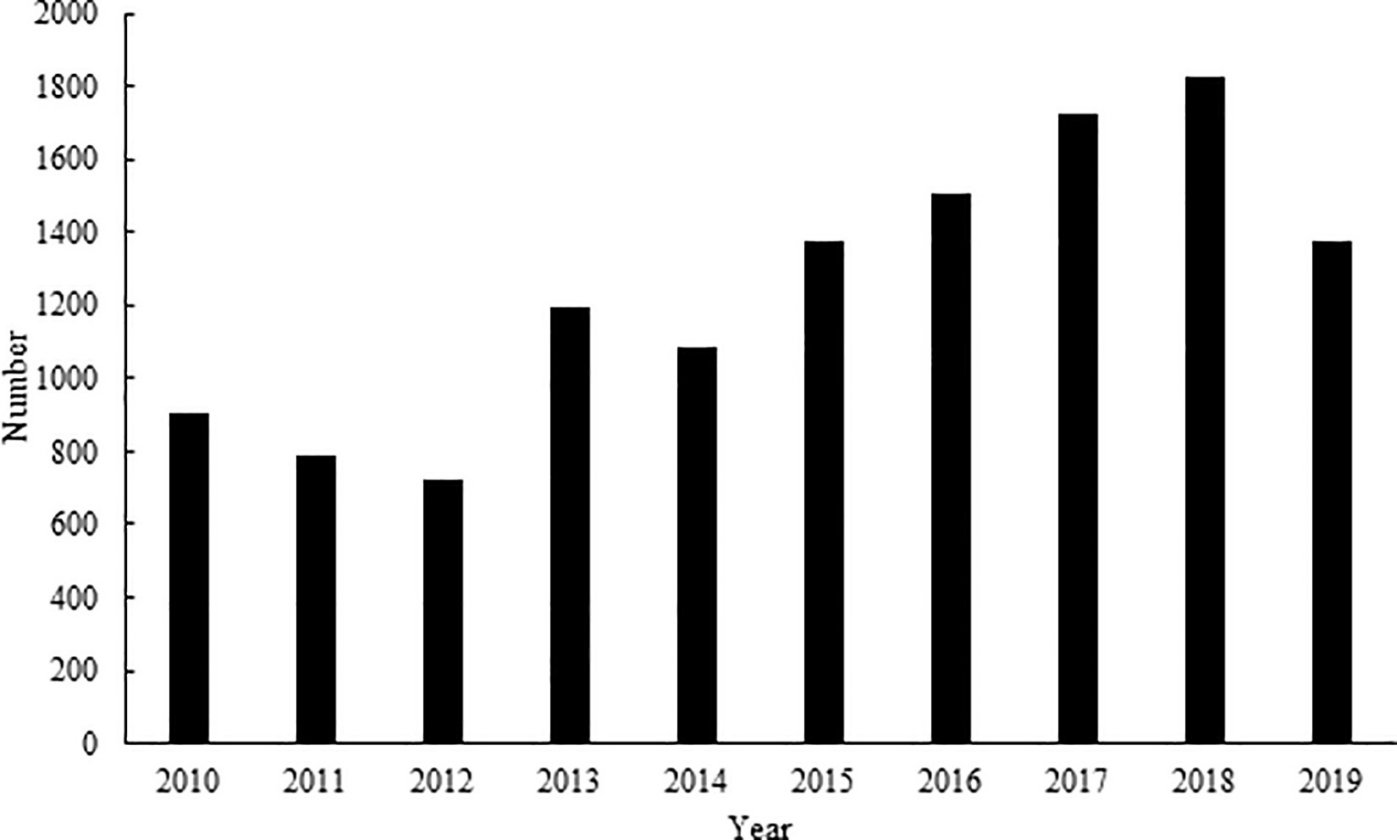
The number of juvenile European hedgehogs admitted to rescue centers in the Czech Republic in the period from 2010 to 2019.

A total of 12,514 juvenile European hedgehogs were admitted to 34 rescue centers in the Czech Republic in the period from 2010 to 2019. The number admitted in individual years is depicted in Fig 1. An increase is evident in the figure in the years 2014 to 2018 in particular. A rising trend in the number of juvenile European hedgehogs admitted to rescue centers in the Czech Republic in the studied period was found (rSp = 0.8667, p < 0.05), despite a slight decline in 2019.

Table 1 shows the number and percentage of juvenile European hedgehogs by the month in which they were admitted.

**Table 1:** Number and percentage of the total number of juvenile European hedgehogs admitted to rescue centers by individual months in the period from 2010 to 2019.

	Admitted hoglets (n = 12,514)	
Month	number	%
January	189	1.51^h^
February	82	0.66^i^
March	43	0.34^e^
April	64	0.51^e^^,^^i^
May	120	0.96^d^
June	293	2.34^f^
July	379	3.03^g^
August	1,863	14.89^k^
September	3,489	27.88^a^
October	3,065	24.49^b^
November	2,043	16.33^c^
December	884	7.06^j^

^a-k^The values with different superscript letters in a column are significantly (p < 0.05) different (Fisher´s exact test).

The largest numbers of juvenile European hedgehogs were admitted to rescue centers in the autumn in September (27.88%) and October (24.49%). The months with the lowest number of admissions were March (0.34%), April (0.51%) and February (0.66%), i.e. the end of winter.

Data on weight on admission was available for 5,304 juvenile European hedgehogs admitted to rescue centers, making it possible to analyse the effect of weight on the release rate and the length of time spent at rescue centers. Table 2 shows the number of juvenile European hedgehogs admitted to rescue centers in the Czech Republic in individual weight categories (for those hedgehogs whose weight was known) and the proportion of those released back into the wild in the studied period of 2010 to 2019. Overall, around a half of the juveniles admitted could be subsequently released (49.38%), with the highest release rate seen in the categories of hedgehogs weighing 400–499 g (63.31%) and 500–599 g (64.22%) and the lowest release rate in hedgehogs weighing 200–299 g on admission to rescue centers (35.24%).

**Table 2:** Number of juvenile European hedgehogs admitted to rescue centers depending on weight and release rate.

Weight (g)	Number of admitted	Number of released	% of released
up to 99	598^t^	291	48.66^a^
100–199	1,564^u^	886	56.44^b^
200–299	1,155^v^	407	35.24^c^
300–399	1,017^w^	465	45.72^d^
400–499	477^x^	302	63.31^e^
500–599	204^y^	131	64.22^f^
600–699	149^z^	85	57.05^g^
700–799	140^z^	52	37.14^g^
**Total**	**5,304**	**2,619**	**49.38**

^a-g^The values with different superscript letters in a column (% of released) are significantly (p < 0.05) different (Fisher´s exact test).

^t-z^The values with different superscript letters in a column (number of admitted hedgehogs) are significantly (p < 0.05) different (Fisher´s exact test).

**Fig 2:**
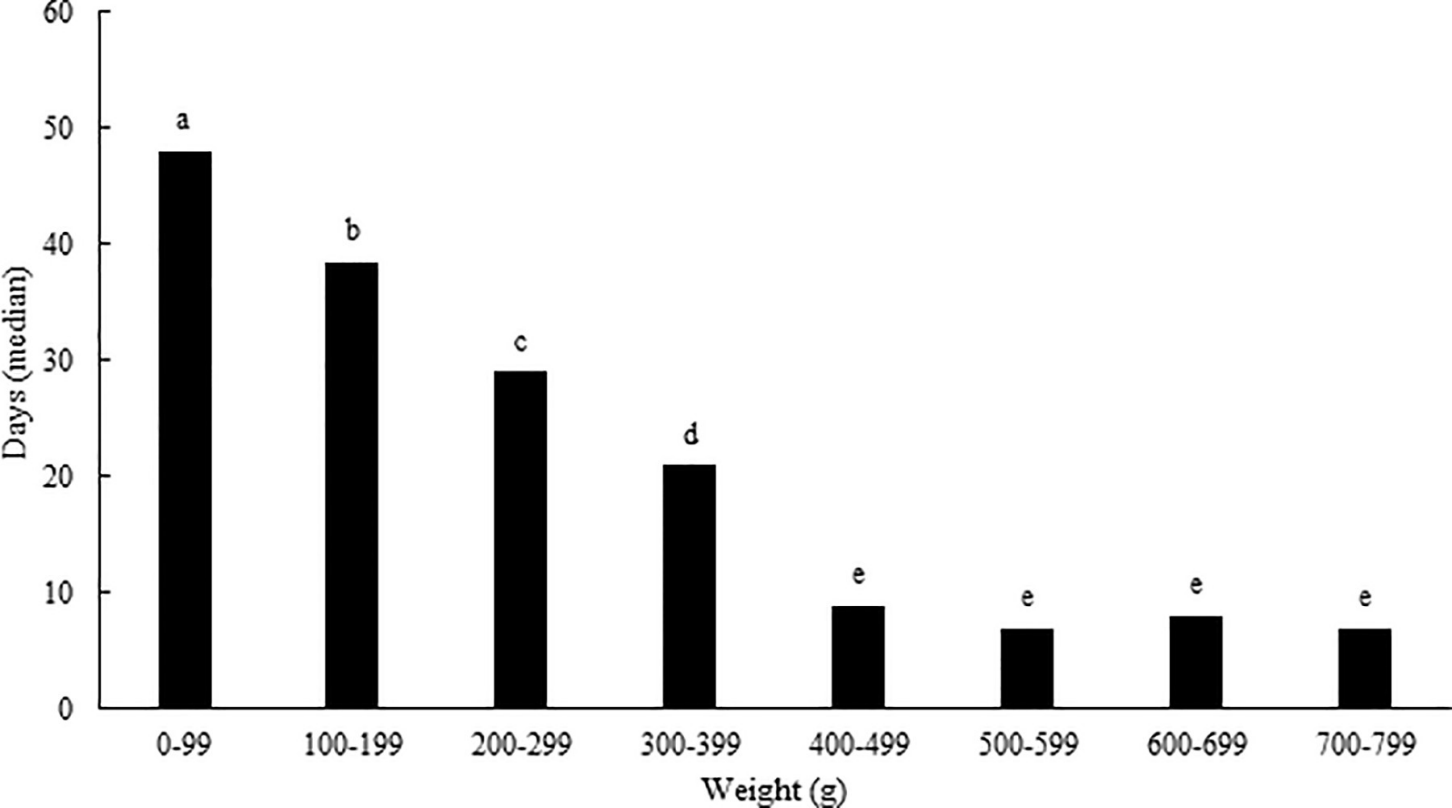
Median of time for individual weight categories of juvenile European hedgehogs released from rescue centers in the Czech Republic back into the wild in the period from 2010 to 2019. * ^a-e^ different letters indicate a statistically significant (p < 0.05) difference (Kruskal-Wallis ANOVA).

The length of stay at the rescue center before release was also evaluated depending on the weight in 2,619 juveniles with known weight at the time of admission (Fig 2).

Juvenile European hedgehogs weighing 400 g and more spent a statistically significantly shorter period at rescue centers than juveniles in lower weight categories. The longest period was spent at rescue centers by young animals weighing up to 99 g (median 48 days) before they could be released. A decreasing trend (rSp = - 0.4738, p < 0.001) was found in the number of days spent at the rescue center with the increasing weight of young.

Table 3 shows the average, median, the minimum and maximum length of time spent at rescue centers in individual weight categories.

**Table 3:** Minimum, maximum, median and average length of stay (days) of juvenile European hedgehogs released from rescue centers in the period from 2010 to 2019.

	Length of stay (days)			
Weight (g)	Minimum	Maximum	Average	Median
up to 99	1.0	236	60.78	48.0
100–199	1.0	232	53.39	38.5
200–299	1.0	239	43.26	29.0
300–399	1.0	192	35.53	21.0
400–499	1.0	210	24.01	9.0
500–599	1.0	195	19.09	7.0
600–699	1.0	175	23.90	8.0
700–799	1.0	372	27.28	7.0

While the minimum number of days spent at rescue centers is the same in all categories, the longest stay on average was seen in hedgehogs in lower weight categories (up to 99 g and 100–199 g).

Table 4 depicts categories of hedgehogs by weight and the percentage of juveniles admitted to rescue centers needlessly and juveniles truly requiring human care. The results indicate that the majority of hedgehogs in the lowest weight categories were admitted for reasons that truly demanded human care for their survival, while the proportion of hedgehogs taken to rescue centers needlessly increased with their increasing weight.

**Table 4:** Percentage of hoglets unnecessarily brought and those requiring human care admitted and subsequently released from the rescue centers depending on weight.

Weight (g)	Reason for admission					
	no apparent reason (%)	orphaned, exhausted or late-born (%)	injuries (%)	infection, parasites (%)	fall into pits etc. (%)	other reasons (%)
up to 99	1.72	92.76	4.14	0.00	1.38	0.00
100–199	1.84	92.18	4.02	0.23	1.49	0.23
200–299	6.09	82.23	2.03	0.51	2.03	7.11
300–399	7.28	73.51	1.77	1.32	4.86	11.26
400–499	27.21	49.12	6.36	1.77	4.24	11.31
500–599	35.45	39.09	2.73	4.55	1.82	16.36
600–699	28.57	19.48	14.29	7.79	10.39	19.48
700–799	32.60	8.70	19.57	6.52	6.52	26.09

## Discussion

The number of European hedgehogs admitted to rescue centers has been increasing over the last ten years [11] and a similar trend can also be seen in many other animal species [24–27]. Various studies have also found an extremely high rate of admission of young animals to rescue centers [28,29]. This fact may be explained in several ways. Increasingly intensive anthropogenic impact on the natural world and on changing the landscape and its use by animals may play a part in the increasing number of animals admitted [30]. Another aspect may be the fact that the public is now better acquainted with the issue of permanently disabled animals and is making greater efforts to help these animals. Unfortunately, these efforts sometimes have the opposite effect in the case of animals, and young animals, in particular, being taken needlessly from their natural habitat [11].

Our results show that the largest numbers of juvenile hedgehogs were admitted to rescue centers in the Czech Republic in the autumn months, i.e. at the time they are preparing for hibernation. Hedgehogs lose body weight during hibernation [31] and low body weight before hibernation begins may result in the death of a hedgehog due to insufficient energy reserves needed to survive this period. The public might be aware of this risk but the average member of the public can hardly distinguish between juveniles and adult hedgehogs, and concerns about hedgehogs found in the autumn may lead to their being taken to rescue centers even if they do need human care. In contrast, the lowest number of hedgehogs admitted in our study was seen in the period from February to April. In the winter and spring months, these may be individuals found out in the open during cold weather. This occurs rarely, however, as hedgehogs are still hibernating in the winter months and at the beginning of spring when temperatures are not suitable for them [32] and they appear only very rarely in gardens and other open spaces [21,33]. Young hedgehogs admitted to rescue centers in the spring months, i.e. at the time when hibernation is coming to an end, may be animals born in the year in question, although because of their reproductive strategy and the birth of young this tends to occur later in the spring when conditions are more suitable for reproduction [18].

Rescue centers endeavour to release admitted animals back into the wild as soon as possible, i.e. as soon as this is possible from the viewpoint of the condition of the animal and the environmental conditions. The length of stay at the rescue center generally corresponds to the period required for convalescence and the attainment of optimal condition for release. Young animals up to 99 g in weight spent the longest period at rescue centers in the Czech Republic (median 48 days) during the monitored period even though the success rate from the viewpoint of their release was not high (just 48.66% of young in this weight category were subsequently released). In contrast, of all the categories the release rate was highest (64.22%) in hedgehogs weighing 500–599 g, which also spent the shortest period at rescue centers (median 7 days). The results indicate that weight and condition on admission have a greater influence on survival and the ability to return to the wild in juvenile European hedgehogs than the length of time spent at the rescue center and the care provided. This corresponds to the fact that the majority of young weighing up to 99 g (98.28%) were brought to rescue centers because they were truly abandoned, malnourished or otherwise handicapped (injured, diseased etc.). In contrast, a life-threatening handicap was found in only 64.55% of hedgehogs weighing 500–599 g when admitted to rescue centers, while 35.45% of young in this weight category were taken to rescue centers needlessly. These animals did not require any care and could be returned to the wild in a short time. As the percentage of the released young animals in this category shows, however, a large number of those that needed care were also successfully treated and prepared for release. In the smallest hedgehogs, in contrast, the attempt to save and raise them often failed and a higher mortality rate was recorded in this category. Care for these young animals at rescue centers consists primarily of providing suitable conditions and correct nutrition and minimising stress. Stress, in particular, can have a negative effect on the possibilities for the convalescence and rearing of young and their release back into the wild, as it has a great effect on processes in the organism and its resilience [34]. It is not always particularly easy to raise young on a milk diet properly given the quality of milk, and this is a problem in many animal species [35]. The young may also be non-viable from birth in some way, for which reason they have been abandoned by their mother [36]. Another reason for the differing success rates in the rearing of young hedgehogs at rescue centers may be the greater resilience of older young to the stress to which they are exposed at rescue centers in the proximity of humans or their ability to survive hibernation at rescue centers.

It is difficult to tell with any certainty whether juvenile European hedgehogs are young from the first or second litter of the given year because of the difficulty of determining their age, and it is, therefore, hard to estimate what chance a given individual has of successful rearing or survival at a rescue center. Pregnancy in female European hedgehogs lasts an average of 35 days, with the first litter born in May or June and any second litter in August or September [37], though these dates are of only limited applicability and there is also always the possibility of a delayed first litter in the case of a female who has lost her first young. Several other factors may also have an effect on the weight of hedgehogs during the year, such as the period after hibernation and mating [38]. The weight categories in this study reflect the age of the hedgehogs to a certain extent. According to Haigh et al. [23], a correlation exists between weight and other parameters according to which the approximate age of a hedgehog may be determined. Deanesley [39] reported the onset of puberty in female hedgehogs at a weight of around 400 g, and this weight category can be considered the period of adolescence. Mullineaux [40] based a division into age categories according to certain parameters such as the state of the teeth and the appearance of the spines and also reported the weights typical of these age categories. According to that young are born at a weight of 12–20 g and attain a weight of 100 g at the age of 3 weeks and a weight of 200 g at the time of weaning which is in around the 5th or 6th week of life. According to this division, the category of hedgehogs weighing up to 99 g would be young that have so far been on a milk diet, while hedgehogs weighing 100–199 g would be hedgehogs at the time of weaning and the transition to solid food and an independent way of life. Hedgehogs weighing around 400 g are adolescent individuals, while those weighing 600–800 g are young adult individuals. According to Kristiansson [41], the availability of food and environmental factors have a greater influence than age as far as the mortality rate in young hedgehogs in the wild is concerned. According to our results, however, weight is a fundamental factor influencing the success of care for juvenile hedgehogs at rescue centers. Juveniles up to the weight of 200 g, i.e. young on a milk diet and at the time of weaning, demand specific care that rescue centers are often incapable of providing, in contrast to adolescent and adult hedgehogs.

The length of stay at rescue centers differed in the individual weight categories, with the longest period (median of days) seen in the youngest weight categories, which reflects the fact that these young require longer care until they are capable of looking after themselves and being released into the wild. We attribute the minimum length of stay of one day primarily to cases in which young were admitted to rescue centers needlessly with no evident health problem and were subsequently released back into the wild, or re-joined with their mother in the case of unweaned young, after being examined by rescue center staff. They may also have been animals from nests threatened by destruction, for example. Such cases occurred in all weight categories. The average length of time spent at rescue centers differed significantly depending on their weight at the time of admission in those hedgehogs that stayed for longer periods. Differences were also found in the percentage of hedgehogs released in individual weight categories. The lowest success rate from the viewpoint of the percentage of animals released (35.24%) was, surprisingly, found in the category young admitted at a weight of 200–299 g, i.e. probably at the time of weaning. These young should no longer be so dependent on human care, and their nutrition in captivity is not as demanding as it is in young on a milk diet for which the percentage of animals released was, however, significantly higher (56.44%). This result may confirm the assumption that the weaning period is a critical time for wild animals, and even human care need not help individuals survive this period, while certain other circumstances such as the death of the mother and premature weaning may reduce the chance of survival of these young still further [42]. Moreover, young animals admitted in an impaired state of health predominated significantly in this weight category. However, even in the 700–799 g category, only 37.14% of hedgehogs were released. Most of these hedgehogs (67.40%) were admitted due to injuries, infections, exhaustion or fall into pits, thus the low release rate in this category may result from the consequences of their impaired health rather than the fact that almostadult animals could not take care of themselves. Overall, it can be said that the number of releases was higher in hedgehogs of greater weight on admission, though the differences were not as high as might have been expected. Overall, it proved possible to release less than a half of the juvenile hedgehogs and the proportion of animals released did not exceed 65% even in hedgehogs whose weight indicated adolescent or almost adult animals.

From the viewpoint of evaluating the success of the work of rescue centers, it is important not merely how many admitted animals can be released back into the wild, but also how many of them than survive in the wild and are capable of reproducing, i.e. actually contributing to maintaining the population of the given species. Preparing them for life in the wild is no less important from this perspective since young animals brought to rescue centers during the first days of life will not necessarily be capable of looking after themselves in the future. Selecting the ideal release site is the final step in the survival of reared young, though the most important. There have been a few studies devoted to survival rates in animals following release from rescue centers. The survival of hedgehogs released from rescue centers seems to be relatively high [43], even in the case of hedgehogs released during the hibernation period [20]. Rasmussen et al. [20] found 59% of females and 79% of males surviving their first hibernation in Denmark. Similar results were reported by Kristiansson [41] in Sweden. The mortality of hedgehogs during or after hibernation in the wild is more often a result of factors connected with human activity (e.g. mortality on the roads) and predation, rather than an inability to look after themselves [33,44]. Rautio et al. [45] also reported road mortality as the most common cause of hedgehog mortality in the wild in Finland. The time spent at a rescue center may be beneficial for injured or diseased hedgehogs [46]. Natural patterns of behaviour need to be preserved in juvenile European hedgehogs reared at rescue centers although the time spent in the rescue center does not appear to have a negative impact on natural patterns of behaviour [21]. The time of release also plays a role in addition to the site of release, though it is thought that releasing hedgehogs in the autumn or spring poses no problem. Still, the period following hibernation is risky [21]. In addition to the possibilities for rearing juvenile European hedgehogs and the factors that reduce or increase mortality in the lowest weight categories, further research must also be devoted to maximising their survival in the wild following their release from rescue centers.

## Conclusion

Juvenile European hedgehogs are often admitted to rescue centers and their survival may depend on many factors preceding their admission as well as on the care given at the rescue center. The results of our study indicate differing success rates in the rearing of hedgehogs admitted at various ages (weights) and differing periods required for their rehabilitation. Rearing the youngest categories appears to be particularly problematic with the lowest release rate (35.24%) found in hedgehogs weighing 200–299 g on admission to rescue centers. However, the release rate was not much higher even in the heavier categories. From this perspective, the finding that hedgehogs that are thought to be young that are not yet independent, though they are in reality adolescent or adult individuals that do not require human care, are often brought to rescue centers is also alarming. Members of the public who want to provide help to animals often cannot correctly identify hedgehogs that truly require human care. The results of this and other studies indicate that even animals brought to rescue centers healthy and in good condition need not necessarily cope with conditions in captivity, for which reason it is essential to eliminate such cases of wild animals being brought to rescue centers unnecessarily as much as possible.
